# Safety and effectiveness of the da Vinci robot with the "3+2" mode for distal pancreatectomy

**DOI:** 10.1002/cam4.2353

**Published:** 2019-06-18

**Authors:** Weipeng Zhan, Ming Hu, Caiwen Han, Hongwei Tian, Wutang Jing, Xiaofei Li, Hao Shi, Xiaojun Yang, Tiankang Guo, He Su, Yuntao Ma

**Affiliations:** ^1^ Department of General Surgery Gansu Provincial Hospital Lanzhou China; ^2^ Institution of Clinical Research and Evidence Based Medicine Gansu Provincial Hospital Lanzhou China; ^3^ Key Laboratory of Molecular Diagnosis and Precision Treatment of Surgical Tumors in Gansu Province Lanzhou China; ^4^ Evidence‐Based Medicine Center Lanzhou University Lanzhou China

**Keywords:** “3+2” mode, distal pancreatectomy, pancreatic neoplasm, robotic surgical

## Abstract

**Background:**

Recently, no relevant research has focused on the relationship between the clinical efficacy of da Vinci robotic distal pancreatectomy (RDP) and the number of mechanical arms and assistants used for RDP. The aim of this study was to evaluate the safety, efficacy, and advantages of RDP with the “3 + 2” mode.

**Methods:**

Clinical data from 53 patients (observation group) who received RDP using the “3 + 2” mode in our department, from March 2016 to September 2018, were reviewed. An additional 53 patients who received RDP using the classical mode were chosen at random for the control group. Short‐term outcomes for the two groups were compared.

**Results:**

There were no statistically significant differences between the two groups for estimated blood loss, postoperative day of flatus passage, postoperative hospital stay, and postoperative complication (*P* > 0.05). Compared with the control group, the observation group had a significantly shorter operative time (166.9 ± 13.3 vs 192.6 ± 11.1 minutes, *P* < 0.001), lower surgical costs ($2827.79 ± $173.02 vs $3900.63 ± $317.29, *P* < 0.001).

**Conclusions:**

The RDP using the “3 + 2” mode can increase the exposure of surgical field, improve cooperation between assistants, lower the surgical costs, and shorten the operative time and learning curve. Moreover, the clinical effect is equal to that of RDP using the classical mode. These findings indicate that RDP using the “3 + 2” mode is safe and feasible for institutions that are equipped for robot‐assisted surgery.

## 1 INTRODUCTION

The pancreas is in a deep retroperitoneal position with rich blood supply; this means it is difficult to expose the organ during surgery, which makes pancreatic surgery one of the most complicated abdominal surgeries.^1^, ^2^, ^3^ Recently, laparoscopic distal pancreatectomy (LDP) has achieved outstanding clinical effects due to the development of minimally invasive technique.^4^ However, the anatomy of the pancreas is complicated because of abundant blood vessels. The laparoscopic arms are not flexible enough for traction hemostasis, and suturing in a two‐dimensional view field during pancreatic surgery.^5^ Therefore, the widespread application of LDP presents some challenges.

The introduction of the da Vinci Surgical System (DVSS) partially overcomes the limitations of laparoscopic surgery.^6^, ^7^ The advantages of da Vinci robotic distal pancreatectomy (RDP) are proven, especially for spleen‐preserving pancreatic surgery; its success has been attributed to the high‐resolution three‐dimensional field of view, the highly flexible system, and careful procedures.^6^, ^8^, ^9^, ^10^ Many studies have demonstrated the safety and efficiency of the RDP.^10^, ^11^, ^12^ Nevertheless, the long operative time and high cost restrict further development.^13^, ^14^

Initially, the DVSS was composed of two mechanical arms and one lens arm. Later, the DVSS was developed to comprise three mechanical arms and one lens arm. Currently, most institutions use the da Vinci robot in classical mode (four arms and one assistant) for medical surgeries. However, there has never been a study of the effect of the number of mechanical arms and assistants on the clinical efficacy of RDP. In this study, clinical data from patients who received RDP using the “3 + 2” mode and those who received this surgery using the classical mode were reviewed. Relevant operative indicators under the two modes were compared to evaluate the efficiency, safety, and advantages of the RDP with “3 + 2” mode.

## 2 MATERIAL AND METHODS

### 2.1 Clinical data

Clinical data from 53 patients (observation group) who received RDP in our department with the “3 + 2” mode, from March 2016 to September 2018, were reviewed. An additional 53 patients who received RDP using the classical mode were chosen at random to form the control group. A computed tomography examination was performed and potential difficulties associated with the lesionectomy were evaluated by magnetic resonance imaging or magnetic resonance cholangiopancreatography before the surgery. All surgeries were performed after the patient or their relatives signed the informed consent form, including agreement to the extra surgical expense associated with the robot. This study was approved by the Ethics Committee of the Gansu Provincial hospital.

### 2.2 Operation layout of the da Vinci robot with “3 + 2” mode

The “3 + 2” mode of the da Vinci robot refers to the operation mode, which uses three mechanical arms (two operation arms and one lens arm) and two assistants. In the surgery, the anesthetist and anesthesia monitor were placed at the foot of the patient, and the instrument carriages were placed to the left of the patient (one instrument carriage was equipped with the laparoscope and robot operation instrument, and the other instrument carriage offered standby open operation instruments). The first assistant and instrument nurse stood next to each other to the left of the patient and the second assistant was to the right of the patient. A display was placed at the opposite side of each assistant. The da Vinci robot was placed adjacent to the head of the patient. The operation table was at a fixed corner of the operation room.

### 2.3 Trocar positions for the “3 + 2” mode of the da Vinci robot

**Figure 1:**
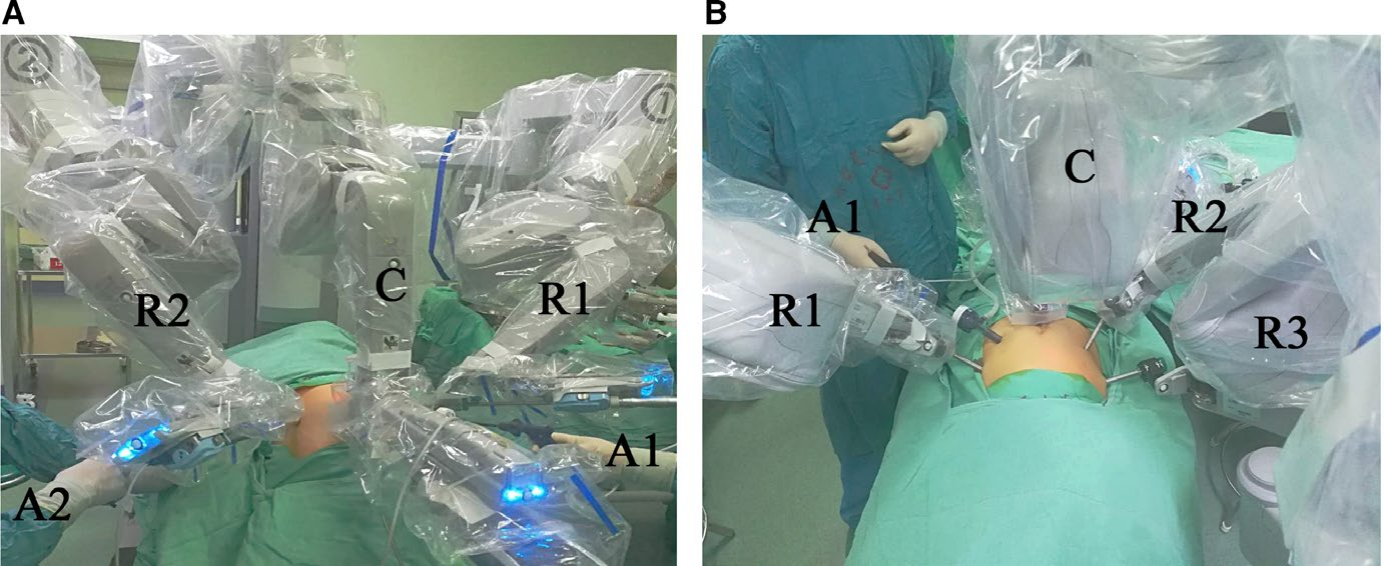
(A) R1, Robotic first arm; R2, robotic second arm; A1, the first assistant; A2, the second assistant; C, the camera arm. (B) R1, Robotic first arm; R2, robotic second arm; R3, robotic third arm; A1, the first assistant; C, the camera arm

The trocar positions for RDP using the "3 + 2" mode were determined, based on our previous investigation and experience. First, a 1.2‐cm long (approximate) longitudinal cut was made 2 cm below the navel, in which a 12‐mm trocar was inserted as the observation port. Pneumoperitoneum was established using CO_2_, the pressure was maintained at 12 mm Hg. Next, an 8‐mm trocar was inserted 2 cm below the costal margin of the left anterior axillary line under direct view, and the robotic first arm (R1) was installed. A 12‐mm trocar was then inserted at the flat navel level of the left midclavicular line; this was used as the first assistant's port site. Similarly, an 8‐mm trocar was inserted at the flat navel level of the right midclavicular line, and the robotic second arm (R2) was installed. A 5‐mm trocar was inserted 1 cm below the costal margin of the right anterior axillary line as the second assistant's port site. All trocar positions were adjustable to suit the body of the patient. The gap between any two trocar positions was controlled to be at least 8 cm to prevent collision of the mechanical arms (Figure 1A).

### 2.4 Surgical technique

#### 2.4.1 Observation group

The DVSS (Intuitive Surgical, Sunnyvale, CA) was utilized. The supine position, with head high and feet low, was applied, forming a body angle of 30°. The right side was inclined by 15‐20°. Body parts that bore stresses were protected from crush damage by a sponge or water cushion. Tracheal intubation was accomplished after general anesthesia induction and the robot operation arm system was installed at the head end of the patient. The Kimura technique^15^ was the first choice for benign and borderline pancreatic neoplasms. The gastrocolic ligament was opened with a piece of an ultrasonic scalpel to expose the tails of the pancreas and hilum of the spleen. Next, the ligaments surrounding the spleen were cut. After moving away the lower position of the pancreas, the operation arm entered into the posterior peritoneum to separate splenic veins from the pancreas from right to left along the pancreas and splenic vein planes, finally arriving at the hilum of spleen. During this process, attention was paid to the ligation at the splenic vein in the pancreatic parenchyma to protect the splenic vein and ensure spleen preservation. Blood vessels at the hilum of the spleen were dissociated. The splenic artery vein was separated from the tail of the pancreas at the hilum of the spleen. The tail of the pancreas was lifted and the part of the splenic artery at the upper edge of the pancreas to the celiac axis was dissociated. Next, the tail of the pancreas was separated completely from the spleen, the splenic arteries, and veins. The pancreas was cut by the Endo‐GIA. The Warshaw technique combined with splenectomy was adopted when it was difficult to separate the tumor or pancreas parenchyma from the splenic blood vessels or when there was bleeding during the separation. The Warshaw technique is similar to the Kimura technique. However, when the tail of the pancreas is difficult to separate from the splenic arteries and veins due to unclear anatomy of the pancreatic lesion and the splenic arteries and veins, a Hem‐o‐lok was used to cut the splenic arteries and veins, while the splenocolic ligaments, gastrosplenic ligaments, and the left blood vessels of the stomach were completely preserved to assure blood supply to the blood vessels at the side of the spleen. Distal pancreatectomy combined with splenectomy should be used in patients with large sized tumors, eggshell calcification, or when there is doubt about canceration. Two assistants cooperated with the operator closely throughout the surgery. The first assistant was responsible for separation, occlusion, and dividing surgeries, as well as discontinuous low‐flow traction using an aspirator and blunt dissection. The second assistant was primarily responsible for traction of organs and providing a sufficient field of view and tension for the surgeon.

#### 2.4.2 Control group

The patient's position and surgical procedure were the same as those of the observation group. First, a 1‐cm long (approximate) longitudinal cut was made 1 cm below the navel, in which a 12‐mm trocar was inserted as the observation hole. Pneumoperitoneum was established using CO_2_, the pressure was maintained at 12 mm Hg. Second, an 8‐mm trocar was inserted 1 cm below the costal margin of the left anterior axillary line under direct view, and the #1 arm of the robot was installed. A 12‐mm trocar was inserted between the hole of the #1 arm and the observation hole, which was used as the surgical hole for the assistants. Similarly, an 8‐mm trocar was inserted 1 cm below the costal margin of the right anterior axillary line and the #3 arm of the robot was installed. Another 8‐mm trocar was inserted between the observation hole and the hole of the #3 arm, and the #2 arm of the robot was installed. All trocar positions could be adjusted to suit the body of the patient, and each trocar was at least 8 cm away from the others to prevent collision of the mechanical arms (Figure 1B). Installation of ultrasonic scalpels, mechanical arms, the lens, and surgical procedures were the same as those in the observation group.

### 2.5 Observation indicators

Observation indicators included operative time, estimated blood loss, postoperative day of flatus passage, postoperative hospital stay, postoperative complications, surgical costs, and total hospitalization costs, spleen preservation rate, 30‐day readmission, and 90‐day mortality. Postoperative complications were graded using the Clavien‐Dindo classification.^16^ The operative time started from skin incision to skin closure, and it excluded the docking and undocking time of robotic surgery. The postoperative hospital stay was defined as the time between the date of surgery and date of discharge.

### 2.6 Statistical analysis

All statistical analyses were conducted using the IBM spss 22.0 software (SPSS Inc, Chicago, IL). Multivariate linear regression models were created using Stata software version 12.0 (Stata Corp, College Station, TX). The data were compared using the chi‐squared test or the Fisher's exact test for categorical variables. The Student's *t* test was applied to compare normally distributed continuous variables, and the Mann‐Whitney U test was used for non‐normally distributed variables. Continuous variables are presented as mean ± SD or as median, interquartile range as appropriate. Confidence intervals (CIs) were estimated at 95%. Values of *P* < 0.05 were considered to be statistically significant.

## 3 RESULTS

### 3.1 Patient characteristics

A total of 106 patients were included in the study. Among them, 53 patients were included in the observation group and other 53 were included in the control group. There were no significant differences in age, sex, body mass index, American Society of Anesthesiologists’ (ASA) score, and tumor size between the two groups (Table 1).

**Table 1:** Characteristics of patient between the two techniques

Patients characteristics	Observation group (n = 53)	Control group (n = 53)	*P* value
Gender (M/F)	35/18	29/24	0.233^a^
Age, median (IQR), y	42 (38‐50)	39 (35‐45)	0.081^b^
BMI, mean (SD), kg/m^2^	24.3 ± 2.0	23.7 ± 1.9	0.112^c^
Largest tumor size, mean (SD), mm	34.3 ± 9.3	32.1 ± 6.6	0.147^c^
Malignancy, n (%)	17 (32.1)	21 (39.6)	0.418^a^
I	2 (3.8)	6 (11.3)	
II	11 (20.8)	14 (26.4)	
III	4 (7.5)	1 (1.9)	
ASA score, n (%)			
1	9 (17)	3 (5.7)	0.158^a^
2	41 (77.4)	45 (84.9)	
3	3 (5.7)	5 (9.4)	

Note

Values are presented as mean (SD) or n (%); median (25th, 75th percentile) as IQR.

a Chi‐squared test.

b Mann‐Whitney test.

c *t* test.

### 3.2 Perioperative outcome

Perioperative outcomes for both groups are shown in Table 2. Surgeries for both groups were accomplished successfully without conversion to laparotomy. One patient in the observation group required a blood transfusion due to an injury to the splenic artery. No patients in the control group required a blood transfusion. The results show that the operative time for the observation group was significantly shorter than for the control group (166.9 ± 13.3 vs 192.6 ± 11.1 minutes, *P* < 0.001). After accounting for confounding factors, the multiple linear regression model also indicated that the control group had a longer operative time than observation group (coefficient = 24.32, 95% CI = 19.28‐29.36; *P* < 0.001) (Table 3). There were no statistically significant differences in the rate of intraoperative blood loss, rate of blood transfusion, conversion rate, and postoperative hospital stay between two groups (*P* > 0.05). Postoperative complications were found in 12 of the 53 patients (22.6%) in the observation group, and in 15 of the 53 control group patients (28.3%). The incidence of complications between the two groups was not statistically different (*P = *0.328). The rate of 30‐day readmission was 9.4% (5/53) in the observation group and 5.7% (3/53) in the control group. No 90‐day mortality occurred between the two groups.

**Table 2:** Surgical outcomes, and short‐term postoperative course and cost analysis

Clinical parameters	Observation group (n = 53)	Control group (n = 53)	*P* value
Operative time, mean (SD), min	166.9 ± 13.3	192.6 ± 11.1	<0.001^c^
Estimated blood loss, median (IQR), mL	90 (70‐155)	110 (80‐175)	0.275^b^
Conversion to open procedure, n (%)	0 (0)	2 (3.8)	0.495^d^
Blood transfusion, n (%)	1 (3.3)	0 (0)	0.315^d^
Postoperative hospital stay, median (IQR), d	12 (10‐14)	11 (9.5‐13)	0.261^b^
Time to passage of flatus, mean (SD), d	2.5 ± 0.6	2.3 ± 0.5	0.089^c^
Postoperative complications, n (%)	12 (22.6)	15 (28.3)	0.328^a^
Clavien 1‐2, n (%)	8 (15.1)	13 (24.5)	0.165^d^
Clavien ≥3, n (%)	4 (7.5)	2 (3.8)	0.339^d^
Pancreatic leakage (%)	2 (3.8)	3 (5.7)	0.647^d^
Chylous leakage (%)	2 (3.8)	1 (1.9)	0.558^d^
Bowel obstruction, n (%)	0 (0)	2 (3.8)	0.248^d^
Wound infection (%)	3 (5.7)	2 (3.8)	0.647^d^
Delayed gastric emptying (%)	1 (1.9)	1 (1.9)	0.752^d^
Pulmonary infection (%)	1 (1.9)	4 (7.5)	0.363^d^
Total hospitalization cost, mean (SD), $	9891.38 ± 675.5	11222.43 ± 904.24	<0.001^c^
Operation cost, mean (SD), $	2827.79 ± 173.02	3900.63 ± 317.29	<0.001^c^
30‐day readmission	5 (9.4)	3 (5.7)	0.462
90‐day mortality	0	0	—

Note

Values are presented as mean (SD) or n (%); median (25th, 75th percentile) as IQR.

a Chi‐squared test.

b Mann‐Whitney test.

c *t* test.

d Fisher's exact test.

**Table 3:** Multivariable linear regression model of factors associated with OT, OC

Factors	Operative time (OT)		*P* value	Operation cost (OC)		*P* value
	Coeff	95% CI		Coeff	95% CI	
Control group^a^	24.32	19.28 to 29.36	<0.001	1089.42	941.28 to 1237.55	<0.001
Female^b^	2.05	−2.83 to 6.94	0.407	0.22	−0.3.62 to 104.05	0.997
Age (y)	−0.08	−0.37 to 0.22	0.606	1.12	−5.21 to 7.41	0.729
BMI (kg/m^2^)	−0.51	−1.79 to 0.78	0.434	−4.56	−31.85 to 22.73	0.741
Largest tumor size (cm)	−0.18	−0.48 to 0.12	0.243	−2.61	−9.09 to 3.89	0.428
Estimated blood loss (mL)	0.02	−0.08 to 0.04	0.199	−0.08	−0.59 to 0.42	0.743
Postoperative hospital stay (d)	—	—	—	—	—	—
Time to passage of flatus (d)	—	—	—	—	—	—
Operative time (min)	—	—	—	−0.84	−5.06 to 3.39	0.695

Abbreviations: Coeff, coefficient; CI, confidence interval; BMI, body mass index.

a In reference to Observation group.

b In reference to males.

### 3.3 Spleen preservation and spleen vessels preservation

The preservation of the spleen and spleen vessels for the two groups is shown in Table 4. The spleen was preserved in a total of 51 patients in the two groups, 27 patients in the observation group and 24 patients in the control group. There was no intention to preserve the spleen in patients with malignant tumors. Excluding those with malignant tumors, the percentage of successful spleen preservation for the two groups was 64.8% and 61.5%, for the observation and control groups, respectively. There were no significant differences in the rates of spleen preservation and splenic vessels preservation between the two groups (*P* > 0.05).

**Table 4:** Preservation of the spleen and spleen vessels between the two groups

Clinical parameters	Observation group (n = 53)	Control group (n = 53)	*P* value
Spleen preservation, n (%)	27 (50.9)	23 (43.4)	0.436
Spleen preservation excluded malignancy, n (%)	27 (75.0)	23 (71.9)	0.493^a^
Spleen vessels preservation, n (%)	15 (28.3)	10 (18.9)	0.253
Spleen vessels preservation excluded malignancy, n (%)	15 (41.7)	10 (31.3)	0.374

Note

Data are shown as n (%). Chi‐squared test was used for between‐group comparison.

a Fisher's exact test.

### 3.4 Pathological outcome

The postoperative pathological outcomes for the two groups are consistent with the preoperative pathological findings. The main pathological types found in the two groups were pancreatic ductal adenocarcinoma (30.2% vs 26.4%) and mucinous cystadenoma (24.5% vs 28.3%). Solid pseudopapillary neoplasm (1.9% vs 11.3%) and acinar cells neoplasm (0% vs 1.9%) were present but uncommon. There was no statistical difference in pathological classification between the two groups (Table 5). Both groups also had similar mean largest tumor size (34.3 vs 32.1 mm; *P* = 0.147).

**Table 5:** Clinicopathologic characteristics of patients between the two groups

Histopathology, n (%)	Observation group (n = 53)	Control group (n = 53)	*P* value
Neuroendocrine tumor, n (%)	9 (16.9)	2 (3.8)	0.833^a^
Mucinous cystadenoma, n (%)	13 (24.5)	15 (28.3)	0.659
Serous cystadenoma, n (%)	6 (11.3)	2 (3.8)	0.135^a^
Solid pseudopapillary neoplasm, n (%)	1 (1.9)	6 (11.3)	0.056^a^
Intraductal papillary mucinous neoplasm, n (%)	5 (9.4)	7 (13.2)	0.382^a^
Acinar cells neoplasm, n (%)	0 (0)	1 (1.9)	0.500^a^
Pancreatic ductal adenocarcinoma, n (%)	16 (30.2)	14 (26.4)	0.666
Others, n (%)	3 (5.7)	6 (11.3)	0.244^a^

Note

Data are shown as n (%). Chi‐squared test was used for between‐group comparison.

a Fisher's exact test.

### 3.5 Cost analysis

The economic analysis is presented in Table 2. The results show that the cost of operation in the control group is higher than that in the observation group ($2827.79 ± $173.02 vs $3900.63 ± $317.29, *P* < 0.001). After adjusting for potential confounding factors based on outcomes, a multivariable linear regression model showed that there was a $1263.95 increase in operation cost for the control group compared with the observation group (*P* < 0.001) (Table 3).

## 4 DISCUSSION

The DVSS is characterized by higher resolution of the field of view, more flexible operation, lower conversion rate, and a shorter learning curve compared with the traditional laparoscope technique.^17^, ^18^ Recently, the application of this robot in pancreatic surgeries has attracted significant attention. However, the complexity and anatomical position of the pancreas, and its rich blood supply make the operation difficult, so the tacit cooperation of the team and full exposure of the surgical field are key to the success of the operation.^19^ The classical da Vinci robot mode for pancreatic surgery uses one assistant and four arms (three operating arms and one camera arm). This mode requires frequent changes of the instrument during the surgery, and offers only a small operating field when there is a shortage of assistants, these factors mean that surgery progresses slowly. The addition of one assistant and a reduction in the number of mechanical arms by one (ie, “3 + 2” mode) produces better outcomes. The “3 + 2” mode can shorten the operation time, reduce the costs of surgery, and shorten the learning curve associated with the robot‐assisted surgery, which is beneficial for the training of young doctors. Additionally, there may be a collision between mechanical arms in thinner patients in the classical robot mode. However, such collisions can be avoided in the “3 + 2” mode.

For the da Vinci robotic surgery system, the surgeon needs the help of an assistant to complete the operation. Due to the complicated pancreatic anatomy and the abundant blood vessels surrounding the pancreas, it is crucial to have sufficient exposure and a clear field of view. This can be achieved by RDP using the “3 + 2” mode. During surgery, the first assistant handled the suction device for clearing the operative field, and provided tactile feedback to the surgeon to decrease the possibility of accidental damage caused by the mechanical arm without feedbacks. The second assistant cooperated with the surgeon to fully expose the operating field and provide tissue tension, thus ensuring that the mechanical arm could complete the surgery successfully. In this configuration, frequent changes of instruments are not required during the surgery, which saves a significant amount of operation time. The average operative time in the control group for this study was 25.7 min longer than that of the observation group. While this difference may not have clinical significance, the average operation time under the “3 + 2” mode might be reduced further as the surgeons accumulate more skills and experience in RDP with the “3 + 2” mode.

The spleen is the largest immune organ in the human body. The immunity and filtering functions of the spleen are vital to health, especially with respect to antitumor and anti‐infection functions.^20^ Therefore, it is crucial to preserve the spleen and its functions in pancreatic surgery.^21^, ^22^ Several studies have suggested that leukocytes and blood platelets are increased after splenectomy, which may cause portal thrombosis.^23^, ^24^ Tumor size, relationship between the tumor and the hilum of the spleen, anatomic variation in the splenic vessels, and local inflammatory infiltration are key determinants of spleen preservation.^25^ In this study, the spleen was preserved in 27 patients in the observation group and 24 patients in the control group. In the “3 + 2” mode, the second assistant provided sufficient exposure of the operation field and tissue tension. The first assistant was responsible for assisting the surgeon in timely clamping blood vessels to reduce intraoperative blood loss and unnecessary damage. While there was no statistical significance in the rate of spleen vessels preservation between the two groups, our team found that the “3 + 2” mode was conducive to the separation of spleen vessels and tended to increase the preservation rate of spleen vessels during the operation. With respect to spleen preservation, our choice of robot‐assisted surgery has obvious advantages in the cases of large tumors, benign or borderline tumors with a deep location, and cases where there is close contact between the tumor and the splenic artery and vein. It has been reported that RDP not only increases spleen preservation rates, but also improves the preservation rate of spleen blood vessels compared to LDP.^6^, ^14^, ^26^

The advantages of the robotic platform over the laparoscopic approach include motion stabilization, the absence of the fulcrum effect, reduction in operator fatigue, three‐dimensional and high‐definition vision, seven degrees of freedom, and improved ergonomics for the surgeon.^27^ Although the da Vinci robot is increasingly being used for various surgeries, the high cost has limited the popularity of robotic surgery to some extent.^14^, ^28^ A reduction in the number of assistants partly alleviates issues related to a shortage of human resources. One of the design objectives of the da Vinci robot was to reduce the dependence of surgeons on assistants, thereby reducing the number of assistants and saving medical resources.^29^ However, advanced medical instruments such as the da Vinci robot are scarce in developing countries such as China, especially in the economically underdeveloped Northwest China. The “3 + 2” mode, in which one robotic arm is replaced by an assistant increases surgical flexibility and reduces the costs associated with adding a robotic arm and the cost of consumables used in constant instrument changes. Yim et al^30^ also concluded that a reduction in the number of mechanical arms is an effective way to lower surgical costs while maintaining a satisfactory clinical effect. In this study, the surgical cost for the control group was $1072.84 higher than in the observation group. Thus, the proposed “3 + 2” mode decreases the economic burden on patients. Further, the role of the first assistant in the “3 + 2” mode is assumed by a senior physician with extensive experience in laparoscopic surgery, and the second assistant is filled by less experienced young resident doctors. This enables young resident doctors to become familiar with the DVSS early in their career, which is conducive to the promotion and application of robotics in China. However, depending on the circumstances, these considerations may not be relevant in some developed countries or in hospitals where doctors are scarce. The cost of assistants in some developed countries is higher than the cost of robotic arms. Therefore, the “3 + 2” model is more appropriate for those developing countries or institutions with the purpose of increasing the experience of robotic assistants, such as China.

There are several limitations to this study. (a) As a retrospective study, all data regarding patient demographics as well as intra‐ and postoperative outcomes were retrospectively collected from medical records—this non‐randomized study design is subject to inherent selection bias. However, the surgery in both groups was performed by the same surgical team and there were no statistical differences in the important baseline clinicopathologic characteristics that may affect surgical outcomes. (b) There was no long‐term follow‐up of patients, so the long‐term clinical effects of the RDP with the “3 + 2” mode cannot be evaluated. (c) The "3 + 2" mode da Vinci robot was used only for a short period of time in DP, so the number of cases was not sufficient to produce statistically significant results for all of the outcomes of interest. Hence, it is possible that there may be some false negative results in the study.

To summarize, the “3 + 2” mode of the RDP has an equivalent clinical effect to classical mode RDP with the added benefits of enhanced exposure of the operating field, strengthened cooperation between assistants, reduced surgical costs, and shorter operative time and learning curves. The RDP with the “3 + 2” mode is safe and feasible for surgical institutions that are newly equipped with robot‐assisted surgery. Improvements in the performance of RDP with the “3 + 2” mode might be achieved as the surgeons accumulate more skills and experience. Nevertheless, the long‐term clinical effects of the RDP with the “3 + 2” mode require further verification through high‐quality randomized controlled trials.

## CONFLICT OF INTEREST

Weipeng Zhan, Ming Hu, Caiwen Han, Hongwei Tian, Wutang Jing, Xiaofei Li, Hao Shi, Xiaojun Yang, Tiankang Guo, He Su, Yuntao Ma have no conflict of interest or financial ties to disclose.

## AUTHOR CONTRIBUTIONS

Weipeng Zhan, Ming Hu, Caiwen Han and Yuntao Ma conceptualized and designed the study. Caiwen Han, Hongwei Tian, Wutang Jing, Xiaofei Li, and Hao Shi acquired the data. WeipengZhan, MingHu, and Caiwen Han analyzed and interpreted the data. MingHu, Caiwen Han, and Xiaojun Yang drafted the article. Hongwei Tian, Tiankang Guo, He Su, and Yuntao Ma critically reviewed the article. He Su and Yuntao Ma approved the final article. All authors agree to be accountable for all aspects of the work in ensuring that questions related to the accuracy or integrity of any part of the work are appropriately investigated and resolved.
